# Neural mechanism of circadian clock-based photoperiodism in insects and snails

**DOI:** 10.1007/s00359-023-01662-6

**Published:** 2023-08-18

**Authors:** Yoshitaka Hamanaka, Masaharu Hasebe, Sakiko Shiga

**Affiliations:** Department of Biological Sciences, Graduate School of Science, Osaka University, 1-1 Machikaneyama-cho, Toyonaka, Osaka 560-0043 Japan

**Keywords:** Photoperiodic response, Circadian clock, Neurosecretory cell, Neuropeptide, Clock gene

## Abstract

The photoperiodic mechanism distinguishes between long and short days, and the circadian clock system is involved in this process. Although the necessity of circadian clock genes for photoperiodic responses has been demonstrated in many species, how the clock system contributes to photoperiodic mechanisms remains unclear. A comprehensive study, including the functional analysis of relevant genes and physiology of their expressing cells, is necessary to understand the molecular and cellular mechanisms. Since *Drosophila melanogaster* exhibits a shallow photoperiodism, photoperiodic mechanisms have been studied in non-model species, starting with brain microsurgery and neuroanatomy, followed by genetic manipulation in some insects. Here, we review and discuss the involvement of the circadian clock in photoperiodic mechanisms in terms of neural networks in insects. We also review recent advances in the neural mechanisms underlying photoperiodic responses in insects and snails, and additionally circadian clock systems in snails, whose involvement in photoperiodism has hardly been addressed yet. Brain neurosecretory cells, *insulin-like peptide*/*diuretic hormone44-*expressing pars intercerebralis neurones in the bean bug *Riptortus pedestris* and *caudo-dorsal cell hormone*-expressing caudo-dorsal cells in the snail *Lymnaea stagnalis*, both promote egg laying under long days, and their electrical excitability is attenuated under short and medium days, which reduces oviposition. The photoperiodic responses of the pars intercerebralis neurones are mediated by glutamate under the control of the clock gene *period*. Thus, we are now able to assess the photoperiodic response by neurosecretory cell activity to investigate the upstream mechanisms, that is, the photoperiodic clock and counter.

## Introduction

Photoperiod is the most reliable environmental cue for determining calendar times. From unicellular algae to highly organised vertebrates, a variety of organisms exhibit photoperiodism for seasonal adaptation in middle to high latitudinal regions. The dinoflagellate *Gonyaulax polyedra* enters the dormant stage of the cyst within a short period (Balzer and Hardeland 1991). Similarly, the green alga *Chlamydomonas reinhardtii* enhances the germination efficiency of zygospores on long days and suppresses it on short days (Suzuki and Johnson 2001). The photoperiodic control of reproduction has been reported in different types of birds and mammals (Nelson et al. 2010). In primates, lemurs and Formosan rock macaques respond to the photoperiod for their sexual behaviour or breeding (Perret and Schilling 1995; Heldstab et al. 2021).

In invertebrates, photoperiodism has been reported in Arthropods, especially in Insecta and Mollusca (Saunders 1982; Numata and Udaka 2010). Photoperiodism prevails in insects and controls the mechanisms of seasonal reproduction, growth, and diapause, which have been well studied. In contrast, photoperiodic responses have been studied in a relatively small number of molluscan species. Since the first report of the photoperiodic control of oviposition in the marsh pond snail *Lymnaea palustris* (Jenner 1951), several molluscan species have been shown to display photoperiodism in a wide range of phenomena, including reproduction, growth, cold tolerance, and learning capabilities (Wayne 2001; Flari and Edwards 2003; Koene 2010; Numata and Udaka 2010; Hussein et al. 2020). To date, photoperiodism in Mollusca has been mainly observed in species belonging to Plumonata, Gastropoda (Numata and Udaka 2010). Importantly, even in a marine mollusc of the sea hare *Aplysia californica* (Gastropoda, Opisthobranchia), in which the seawater temperature is considered the most reliable seasonal cue, the photoperiod is still capable of affecting seasonal breeding, although its effect is rather weak (Wayne and Block 1992). This suggests that photoperiodism is a prevailing phenomenon in Gastropoda.

Insecta and Gastropoda are ideal subjects for studying the neural mechanisms underlying photoperiodism in context of the simple nervous system. Photoperiodic mechanisms entail the discrimination of short and long days and the storage of photoperiodic information. These invertebrates are suitable study materials for addressing interesting neurobiological questions.

It is widely accepted that the circadian clock is involved in the time measurement discriminating between long and short days. Bünning (1936) first demonstrated that the flowering of a short-day plant, the scarlet runner bean *Phaseolus coccineus*, is delayed by light exposure during the night phase of the endogenous (circadian) rhythm. Through the great advancement in understanding the molecular machinery of the circadian clock system in which transcription and translation feedback loops of different clock genes time a 24-h cycle, the involvement of these genes has been demonstrated in the photoperiodic responses in different insect species (Saunders and Bertossa 2011; Goto 2023).

**Fig. 1:**
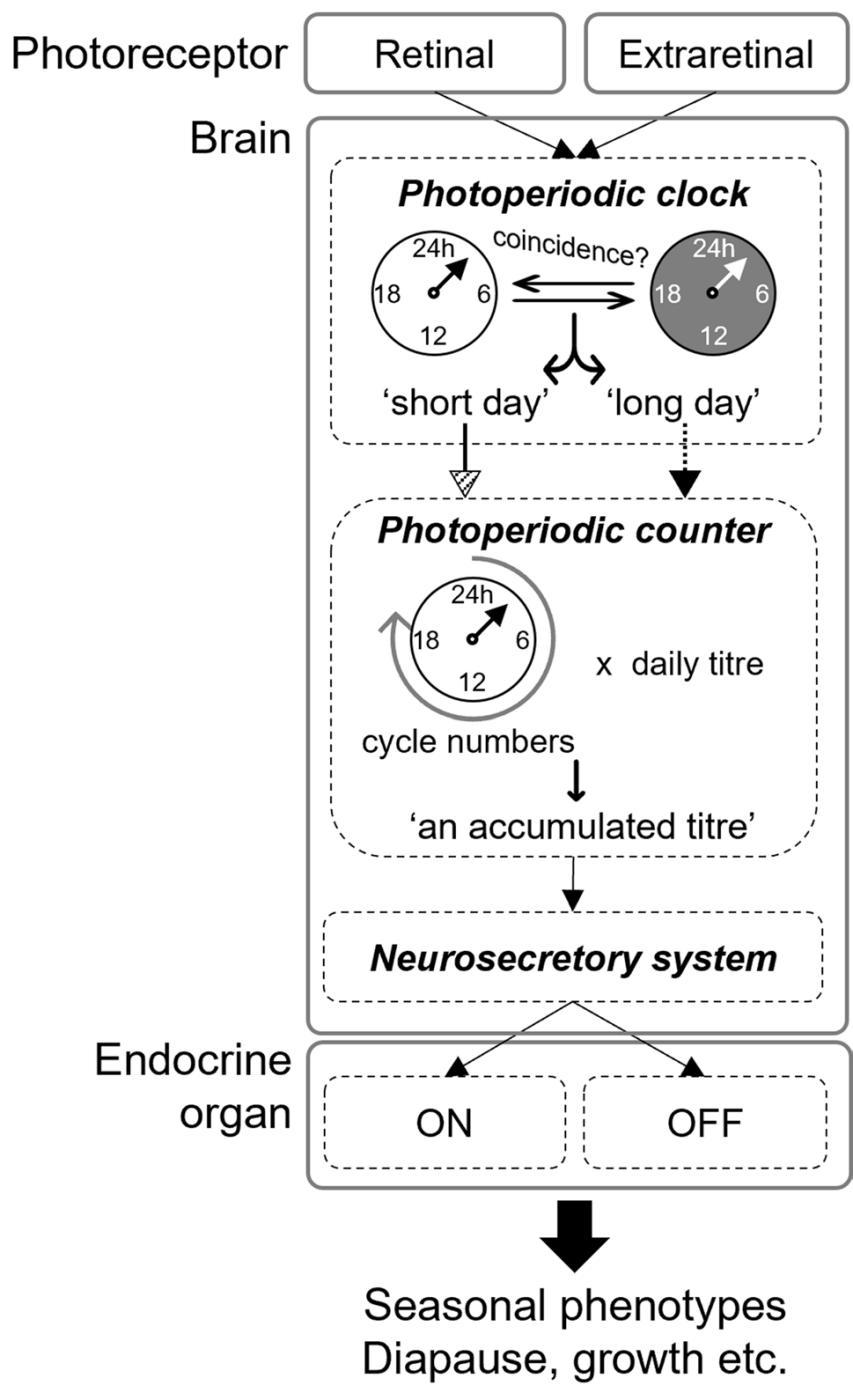
Scheme of potential physiological mechanisms underlying circadian clock-based photoperiodic responses in insects and snails. The circadian clock system is employed in the photoperiodic clock and photoperiodic counter. See text and Fig. 2b for details

Figure 1 shows the postulated circadian clock-based mechanism underlying the photoperiodic responses, which is partly based on the work of Saunders (2002). The mechanism involves photoreceptors, the photoperiodic clock and counter in the brain, and endocrine organs (Fig. 1). The photoperiodic clock, which measures the length of a day, and the photoperiodic counter, which accumulates the necessary number of days, comprise the core photoperiodic mechanism (Saunders 1982, 2002, 2021; Takeda and Suzuki 2022; Shiga 2023). In insects, crucial transplantation and organ culture experiments using the moths *Antheraea pernyi* and *Manduca sexta* have demonstrated that the photoperiodic mechanism involving the photoperiodic clock and counter resides in the brain (Williams and Adkisson 1964; Bowen et al. 1984b; Takeda and Suzuki 2022). Although this scheme has been proposed and is widely accepted in insects, we consider that it could also be applicable to the molluscan photoperiodic responses in which similar characteristics, such as critical day length and requirement of number of weeks for seasonal responses, are evident (Kitai et al. 2021).

The photoperiodic responses are reportedly disrupted by the knockdown or knockout of circadian clock genes in many insect species, which suggests the involvement of clock genes in the photoperiodic clock or counter. However, it is important to focus on the pleiotropic clock gene functions. Several studies have shown that clock gene expression is necessary for the production of photoperiodic phenotypes in peripheral organs but not in the brain. In the gut of the linden bug *Pyrrhocoris apterus*, the juvenile hormone (JH) receptor methoprene-tolerant (Met) and clock proteins Clock (Clk) and Cycle (Cyc) are required to activate the *Par domain protein 1* gene during reproduction and suppress the clock gene *cryptochrome 2*, which promotes the diapause program (Bajgar et al. 2013). Met and Cyc reportedly dimerise to activate circadian rhythm-dependent gene expression in response to JH, which is a process after the endocrine output of the photoperiodic response, in the mosquito *Aedes aegypti* (Shin et al. 2012). This indicates that circadian clock genes have pleiotropic functions, one of which is to regulate seasonal physiology by acting downstream in peripheral tissues, not in the photoperiodic clock or in the counter (Bradshaw and Holzapfel 2010; Bajgar et al. 2013). Considering the potential pleiotropic roles of clock genes, localisation of clock gene-expressing cells and examination of the local elimination effects of these cells in the brain are important for understanding their functions.

To understand the molecular and cellular mechanisms underlying the photoperiodic mechanism, a comprehensive study, including mathematical modelling, identification, and functional analysis of relevant genes and cells in the brain, is necessary. *Drosophila melanogaster* is an ideal model species that is amenable to in vivo manipulation of neurones and neuronally expressed genes to address their functions (Kazama 2015; Venken et al. 2016). However, *D. melanogaster* exhibits a shallow photoperiodic response only at low temperatures (Saunders and Gilbert 1990; Nagy et al. 2018), and detecting photoperiodic effects excluding thermoperiodic effects under light and dark conditions is sometimes challenging (Anduaga et al. 2018). Therefore, studies on the photoperiodic mechanisms have emerged in non-model insect species that exhibit a clear photoperiodism, starting with brain microsurgery and neuroanatomy. Genetic manipulations have been applied to several insect species.

This review introduces the idea of circadian clock-based photoperiodic mechanisms and the neural mechanisms underlying photoperiodic responses in insects and snails. In contrast to insects, the involvement of the circadian clock system in snail photoperiodism remains unexplored. Thus, we mainly introduce the neural mechanism underlying photoperiodism and circadian clock system in snails, and discuss the potential interaction of both the systems.

## Circadian clock-based photoperiodic mechanisms

The photoperiodic clock measures the day or night length of a 24-h day and determines short or long days (Fig. 1). This is a function of the circadian clock system in certain insects. Experimental evidence for this is provided by the interruption of long-period night experiments or resonance protocols (Nanda and Hamner 1958; Hamner 1960; Bünsow 1960; reviewed by Saunders 1981). In these regimes, the period of the light–dark cycle systematically varies over several multiples of the circadian period, and alternate peaks and troughs of a photoperiodic effect (such as diapause incidence) appear approximately 24 h apart. These are interpreted as manifestations of the underlying circadian rhythmicity driven by the circadian clock. External and internal coincidences are two models that explain photoperiodic time measurements using the circadian clock system (Saunders 2020). The photoperiodic responses of the flesh fly *Sarcophaga argyrostoma* fit well with the external, and parasitoid wasp *Nasonia vitripennis* to internal coincidence (Saunders 1974, 1979). These results strongly suggest the involvement of the circadian clock system in photoperiodic time measurement and discrimination between short and long days by the photoperiodic clock (Fig. 1).

In most insect responses, only one-day information on the day length is not sufficient to change photoperiodic phenotypes, such as diapause, and a certain number of short or long days are required. This suggests the presence of a counter mechanism that accumulates unknown titres up to an internal threshold to trigger photoperiodic effects for a certain number of days during a photoperiod-sensitive period (Saunders 1971, 2002; Tyshchenko et al. 1972). Finally, above an internal threshold, the neurosecretory system starts on or shuts off the endocrine system (Fig. 1). As to the photoperiodic counter, Saunders (1981) called the number of long nights (equal to short days), which were needed to raise the proportion of diapause to 50%, the ‘required day number’, and Goryshin and Tyshchenko (1974) called the threshold quantity of diapause titre the ‘critical information parcel’. According to their models, the diapause titre quantities that accumulate in the photoperiodic counter are a function of the number of days. Interestingly, temperature compensation for the required number of days has been demonstrated in *N. vitripennis*, the knot grass moth *Acronicta rumicis* and aphids’ photoperiodic responses (reviewed in Saunders 1981; Hardie and Vaz Nunes 2001). Temperature compensation is a characteristic feature of circadian clocks. These results suggest that the photoperiodic counter mechanism also employs a circadian clock system (Fig. 1). When an accumulated titre corresponding to day-number or circadian clock cycle multiples of daily titres reaches an internal threshold, the neurosecretory system sends signals to the endocrine system.

Photoperiodic clock mechanisms have been extensively discussed in experiments using sophisticated photoperiodic schedules, and detailed models have been postulated and evaluated to explain circadian clock function in the time measurement system (Saunders 1981, 2020). Regarding the photoperiodic counter, some molecular (mRNA or protein) titres are hypothesised to accumulate towards an internal threshold (Yamaguchi and Goto 2019; Lankinen et al. 2021). However, the daily accumulation process in which some day-counting or circadian clock cycle-counting mechanisms works in the brain remains unclear.

## Neural mechanisms underlying insect photoperiodism

### Possible brain neural networks important for the photoperiodic mechanism

**Fig. 2:**
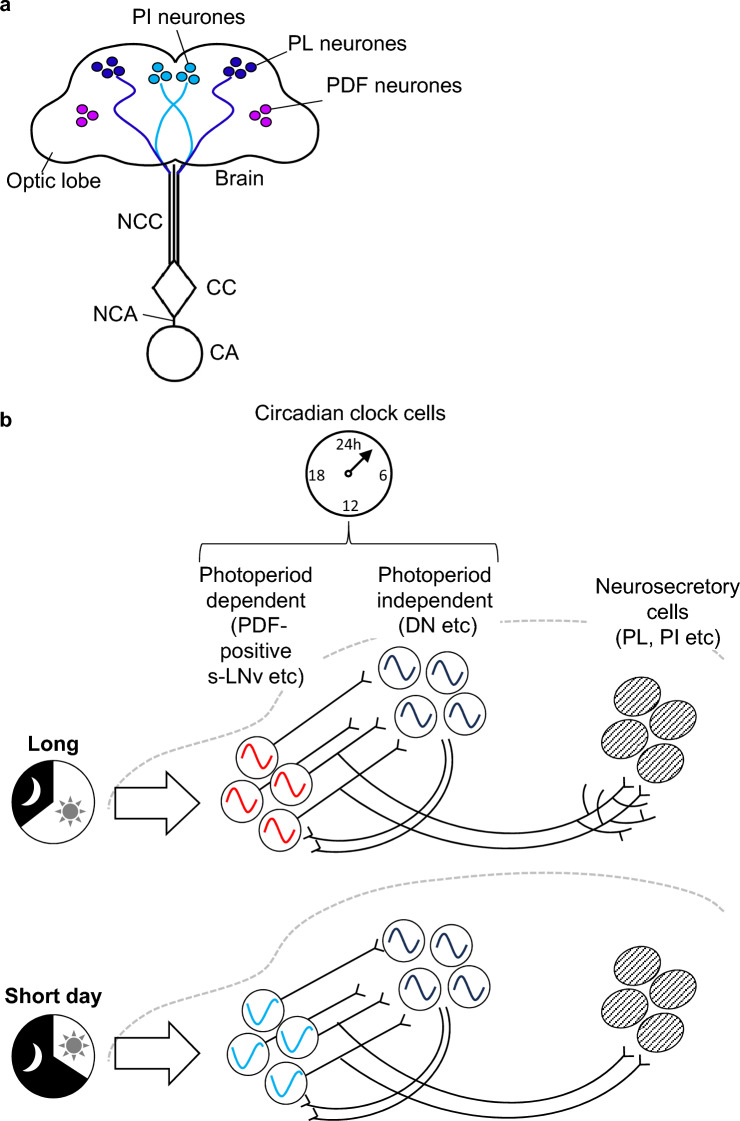
Distribution (**a**) and potential networks (**b**) of circadian clock and neurosecretory cells relevant to the photoperiodic responses in insects. Frontal view. **a** Pars intercerebralis (PI) and pars lateralis (PL) neurones with somata in the dorsal protocerebrum extend fibres to the corpus cardiacum (CC) and corpus allatum (CA) via the nervi corporis cardiaci (NCC) and nervi corporis allati (NCA). Pigment-dispersing factor (PDF) expressing clock neurones locate in the boundary between the optic lobe and mid brain. A type of PDF neurones extend fibres to the dorsal protocerebrum close to neurosecretory PI and PL neurones (not shown). **b** Hypothetical networks of neurosecretory cells and two types of clock cells in the left hemisphere, and their response to long (upper) and short (lower) days. In one type (coloured clock cells), the clock phase depends on photoperiod; in the other type (monochromatic clock cells), it is independent from photoperiod. Accumulation of photoperiodic information may differentiate fibre complexity of photoperiod-dependent clock cells. See text for details

Neuroendocrine systems relevant to photoperiodic responses have been reported in the brain of several species (Shiga and Numata 2007; Barberà et al. 2019). Brain microsurgery and nerve transection experiments suggested that two brain regions, the pars intercerebralis (PI) and pars lateralis (PL), which contain neurosecretory cells (NSCs), are relevant for seasonal phenotypes under photoperiodic control (Fig. 2a, Table 1). Neurones with somata in the PI and PL innervating the endocrine organs of the corpus cardiacum (CC) and corpus allatum (CA) were designated as PI and PL neurones, respectively (Fig. 2a). These neurones release different neuropeptides and amines to neurally or humorally control the endocrine system and other peripheral organs (Raabe 1989; Shiga 2003).

**Table 1:** Effects of different surgeries on the ovarian development in insects

	*Protophormia terraenovae*		*Riptortus pedestris*	
	LD18:6, 25 °C	LD12:12, 20 °C	LD16:8, 25 °C	LD12:12, 25 °C
Intact	+	–	+	–
-NCC	–	–	No data	No data
-NCA	+	+	+	+
-CA	–	No data	–	–
-PI	–	–	+	–
-PL	+	+	+	+
-PDF	± ^a^	±	+	+

Refer to Matsuo et al. (1997), Morita and Numata (1997), Toyoda et al. (1999), Shiga et al. (2000), Shiga and Numata (2009), Shimokawa et al. (2008, 2014), and Ikeno et al. (2014)

+ developed ovary (non-diapause), – immature ovary (diapause), ± diapause incidence was approximately 50%, -*NCC* transection of the nervi corporis cardiaci, -*NCA* transection of the nervi corporis allati, -*CA* removal of the corpus allatum, -*PI* removal of the pars intercerebralis, -*PL* removal of the pars lateralis, -*PDF* removal of regions containing pigment-dispersing factor-immunoreactive cells

^a^Conditions were LD18:6, 20 °C

Another important region is the anterior base of the medulla in the optic lobe where pigment-dispersing factor (PDF)-immunoreactive cells are located (Fig. 2a). In the blowfly *Protophormia terraenovae* and the bean bug *Riptortus pedestris* both of which exhibit a long-day type photoperiodic response controlling ovarian development, PDF-immunoreactive neurones in the optic lobe are necessary for photoperiodic responses. In *P. terraenovae*, PDF co-localises with the circadian clock protein PERIOD (PER) in both the small ventral lateral neurones (s-LNvs) and large ventral lateral neurones (l-LNvs). Elimination of the s-LNvs region caused arrhythmicity in the circadian locomotor rhythm and disrupted the photoperiodic responses, resulting in a 50% diapause incidence, irrespective of the photoperiod (Table 1, Shiga and Numata 2009). In *R. pedestris*, the removal of PDF cell regions at the anterior base of the medulla caused ovarian development on both the long and short days (Table 1, Ikeno et al. 2014). Although PER did not co-localise with PDF in *R. pedestris*, their immunoreactive cells were located in close proximity. This suggests that clock PER cells communicate with PDF neurones in *R. pedeatris* (Koide et al. 2021).

In *D. melanogaster*, PDF abundance in s-LNvs fibres in the dorsal protocerebrum is lower on cold and short days than on warm and long days (Hidalgo et al. 2023). This effect is presumed to be mainly attributed to the temperature rather than the photoperiod. However, it is interesting to note that the PDF produced in s-LNvs regulates seasonal responses through EYES ABSENT (EYA), a co-transcription factor with phosphatase activity (Hidalgo et al. 2023). EYA is expressed in the insulin-producing cells (IPCs) of PI NSCs of *D. melanogaster* and its expression in IPCs is warranted for reproductive dormancy (Abrieux et al. 2020). Here, the interaction between s-LNvs and neurosecretory IPCs was evident (Fig. 2b). In *P. terraenovae*, synaptic connections from s-LNvs to PL neurones, which are necessary for reproductive diapause, have been demonstrated (Hamanaka et al. 2005).

In the *P. terraenovae* brain, different types of clock neurones have been shown to be similar to those in the *D. melanogaster* brain (Shiga and Numata 2009; Kaneko and Hall 2000; Shafer et al. 2006). The logical involvement of the circadian clock system (Fig. 1) and previous brain microsurgery experiments (Shiga and Numata 2009) strongly suggests that some brain clock neurones contribute to the photoperiodic clock and/or counter mechanisms. In *D. melanogaster* approximately 150 clock cells in the whole-brain form neural networks that produce varying behavioural and physiological rhythms (Yoshii and Fukuda 2023). Thus, a subset of clock cells probably calculates day length in the photoperiodic clock and day numbers in the photoperiodic counter in their networks. Interestingly, immunohistochemistry of *P. terraenovae* revealed two types of clock cells in terms of the photoperiodic responses of the nuclear entry timing of PER. When PER nuclear entry was compared from the dark onset, its appearance and disappearance timing appeared similar between short and long days in DNms (corresponding to a type of *Drosophila* DNs) but seemed different in s-LNvs (Fig. 2b; Muguruma et al. 2010; Shiga 2023). In *D. melanogaster*, mutual communication has been reported between s-LNvs and DNs (Fujiwara et al. 2018). In *P. terraenovae*, this interaction may also occur between photoperiod-dependent s-LNvs and photoperiod-independent DNms to integrate photoperiodic information (Figs. 1, 2b). Long- or short-day information may somehow be integrated into the photoperiodic clock mechanism employing s-LNvs and DNms networks and subsequently submitted to the photoperiodic counter. This counter mechanism may also reside in these clock cell networks.

It takes days or weeks to process the day-length signal in the photoperiodic clock and counter system to determine whether the short- or long-day information is solid before changing the neurosecretory system. During this period, the organisms repeatedly receive photoperiodic signals daily. In this counter process, neuronal plasticity may occur in fibre projection or synaptic transmission (Shiga 2013, 2023). In *D. melanogaster*, the dorsolateral protocerebral terminals of s-LNvs show circadian and seasonal plasticity, changing their synaptic partners (Gorostiza et al. 2014; Herrero et al. 2020; Fernandez et al. 2020; Hidalgo et al. 2023). Thus, the accumulation of day or clock cycle numbers may be reflected in complexity or abundance of s-LNv dorsal fibres. As the net intensity of PDF immunoreactivity in the dorsal protocerebrum fibres of *D. melanogaster* s-LNvs decreased under winter conditions (Hidalgo et al. 2023), s-LNv fibres may increase arbours under long-day conditions to alter the signal intensity towards downstream NSCs or alter the target cells between short and long days (Fig. 2b).

Based on the hypothetical concept of a neural network for photoperiodic mechanisms, studies on photoperiodic changes in the neural activities of NCSs and the transmission of photoperiodic information from clock cells to NSCs have recently progressed.

### Photoperiodic changes in the electrophysiological activity of neurosecretory cells

NSCs localised in the PI and PL heterogeneously express various neuropeptides and hormones and are involved in the regulation of various endocrine functions in a paracrine and endocrine fashion (Raabe 1989). The PL contains somata of prothoracicotropic hormone (PTTH)-releasing neurones that release PTTH to activate prothoracic glands and produce ecdysteroids for ecdysis. Their release is suppressed under diapause-inducing photoperiods in lepidopteran species, such as *Manduca sexta*, *Helicoverpa armigera*, and *Mamestra brassicae* (Bowen et al. 1984a; Wei et al. 2005; Mizoguchi et al. 2013). In the PI, the somata of myoinhibitory peptide (MIP) neurones are located in the brown-winged green bug *Plautia stali*. MIPs inhibit JH production in the CA cells, which is presumably suppressed under diapause-inducing conditions (Matsumoto et al. 2017; Hasegawa et al. 2020; Tamai et al. 2019).

Neuropeptides and hormones are packed into large dense-core vesicles (LDCVs), which are typically released by high-frequency action potentials and subsequent increases in the intracellular Ca^2+^ concentration (Hokfelt 1991; Mansvelder and Kits 2000). Excellent physiological studies on the moth *M. sexta* have demonstrated that the release of a neuropeptide eclosion hormone (EH) from NSCs occurs following a surge in spontaneous firing activity, and electrical stimulation of the NSCs can induce EH release (Copenhaver and Truman 1986a, b). These findings suggest a working hypothesis that NSCs in the PI and PL adequately change their firing activity and subsequent neuropeptide/hormone release according to the photoperiod; the latter may contribute to the photoperiodic control of physiological functions. Therefore, elucidating photoperiodic changes in the electrical properties of NSCs is important for understanding the neuroendocrine regulatory mechanisms in photoperiodism. Table 2 summarises the effects of photoperiod on the neuronal activity of NSCs in insects.

**Table 2:** Electrical activities of neurosecretory cells under short- or long-day conditions in insects

Species	Brain region	Cell type/neurotransmitter	Functional role in photoperiodic control of diapause	Conditions	Spontaneous neuronal activity	Photoperiodic difference in neuronal activity	References
*Manduca sexta*							
	PL	PTTH expressing cells	Averting pupal diapause through PTTH release under long days	Short days	Most PTTH cells do not show spontaneous firings	Percentage of spontaneously active neurones increases in long days	Tomioka et al. (1995), Safranek and Williams (1980), Bowen et al. (1984a)
				Long days	25% PTTH cells show spontaneous firing including burst activities		
*Protophormia terraenovae*							
	PI	PIa neurones	Promoting ovarian development under long days	Short days and low temperature	All PIa neurones show high spontaneous firng activities	No differences are observed in electrophysiological properties other than spike duration	Hamanaka et al. (2004), Shiga and Numata (2000, 2007)
				Long days and high temperature	Many PIa neurones show spontaneous high-frequency firings		
	PI	PIb1 neurones	Promoting ovarian development under long days	Short days and low temperature	PIb1 neurones show high spontaneous firing activities	No differences are found in any of electrophysiological properties	Hamanaka et al. (2004), Shiga and Numata (2000, 2007)
				Long days and high temperature			
	PL	PLa neurones	Inducing reproductive diapause under short days	Short days and low temperature	All PLa neurones show high-frequency firings	n.d.	Hamanaka et al. (2004), Shiga and Numata (2000, 2007)
				Long days and high temperature	n.d		
	PL	PLb neurones	Inducing reproductive diapause under short days	Short days and low temperature	PLb neurones show high spontaneous firing activities	No differences are found in any of electrophysiological properties other than spike height	Hamanaka et al. (2004), Shiga and Numata (2000, 2007)
				Long days and high temperature			
*Riptortus pedestris*							
	PI	ILPs/DH44 expressing neurones	Promoting oviposition under long days through ILPs/DH44 release	Short days 5 days after eclosion	Burst, non-burst, and silent activity are found in roughly equal proportions	No differences are found in the spontaneous activity	Hasebe and Shiga (2021a), Shimokawa et al. (2008)
				Long days 5 days after eclosion			
				Short days 20–22 days after eclosion	Most neurones are silent	Spontaneous activities are significantly higher under long-day conditions than under short-day conditions	
				Long days 20–22 days after eclosion	Many neurones show spontaneous activities including burst activities		
*Plautia stali*							
	PI	Plast-MIP/ILP/DH44 expressing neurones	n.d. (Inducing reproductive diapause under short days through Plast-MIP release?)	Short days	Many neurones show spontaneous activities including burst activities	Spontaneous activities are significantly higher under short-day conditions than under long-day conditions	Hasebe and Shiga (2021b), Hasegawa et al. (2020), Matsumoto et al. (2017), Tamai et al. (2019)
				Long days	Many neurones are silent		

*n.d.* not determined, * PI* Pars intercerebralis, *PL* pars lateralis, *PTTH* prothoracicotropic hormone

### Prothoracicotropic hormone neurones in the pars lateralis of *M. sexta*

A pioneering analysis of the photoperiodic effects on the electrical activity of NSCs was performed in PTTH cells in the PL of *M. sexta* (Tomioka et al. 1995). *M. sexta* enters pupal diapause in short days, whereas the moth averts it in long days (Rabb 1966). Pupal diapause under short days is induced by the inhibition of PTTH release from the brain and the absence of ecdysteroid biosynthesis and release (Safranek and Williams 1980; Bowen et al. 1984a). Consistent with these findings, most PTTH cells do not show spontaneous firing activity in short-day-induced diapausing pupae (Tomioka et al. 1995) (Table 2). On the other hand, under diapause-averting long days, 25% of the PTTH cells showed spontaneous firing activity, and high-frequency bursts also occurred, suggesting that PTTH cells upregulate their firing activity under long days and contribute to averting the pupal diapause through PTTH release (Table 2).

### Pars intercerebralis and pars lateralis neurones of *P. terraenovae*

Hamanaka et al. recorded the electrophysiological activities of PI and PL NSCs in adult *P. terraenovae*, injected a fluorescent dye, Lucifer yellow, into the recording cells from a recording electrode, and morphologically grouped the PI and PL NSCs (Hamanaka et al. 2004). PI neurones were divided into three groups: PIa, PIb1, PIb2, and PL neurones, which were also divided into two types: PLa and PLb. All types of PI and PL neurones extend their fibres to the corpus cardiacum–hypocerebral ganglion (CC–HG) complex. Among these, only PIb2 and PLa neurones extended their fibres into the CA. In the fly, the removal of cell bodies in the PL or cutting of the nervi corporis allati (NCA) inhibits diapause induction under short-day conditions (Matsuo et al. 1997; Shiga and Numata 2000, 2007) (Table 1). Thus, PLa neurones that project fibres into the NCA may be strong candidates for inducing diapause over short days.

The electrophysiological activities of PI and PL NSCs were compared under diapause-averting (LH; long day and high temperature) and -inducing (SL; short day and low temperature) conditions (Hamanaka et al. 2004) (Table 2). PIa, PIb1, and PLb neurones showed high-frequency (> 8 Hz) firing activity under both LH and SL conditions; there were no significant differences between the two conditions (Table 2). Although recordings were made from only one PIb2 cell in each condition, the PIb2 cell also showed high firing activity in both the LH and SL conditions. These results indicate that PIa, PIb1, PIb2, and PLb neurones consistently release neuropeptides regardless of the photoperiodic conditions. PLa neurones, which may be important for short-day-induced diapause, were recorded only under diapause-inducing SL conditions and showed high-frequency spontaneous firing activity. This indicates that PLa neurones actively release neuropeptides under diapause-inducing conditions. A recent study on *D. melanogaster* reported that CA-projecting PL neurones (corresponding to PLa in *P. terraenovae*) express the neuropeptide diuretic hormone 31 (DH31), which induces reproductive dormancy by suppressing JH biosynthesis (Kurogi et al. 2023). Based on this report, we hypothesised that PLa neurones photoperiodically alter their neuronal activity and subsequent diapause-inducing neuropeptide (such as DH31) release, contributing to the photoperiodic regulation of diapause induction. Interestingly, immunohistochemistry, combined with retrograde filling from the CA, revealed that a few PL neurones terminating in the CA (possibly corresponding to PLa neurones) exhibited FMRFamide immunoreactivity (Hamanaka et al. 2007). Identification of the diapause-inducing neuropeptides in the PLa neurones of *P. terraenovae* and comparison of PLa neuronal activity between long and short days are essential for understanding the photoperiodic diapause induction mechanisms.

### Pars intercerebralis neurones of *R. pedestris*

**Fig. 3:**
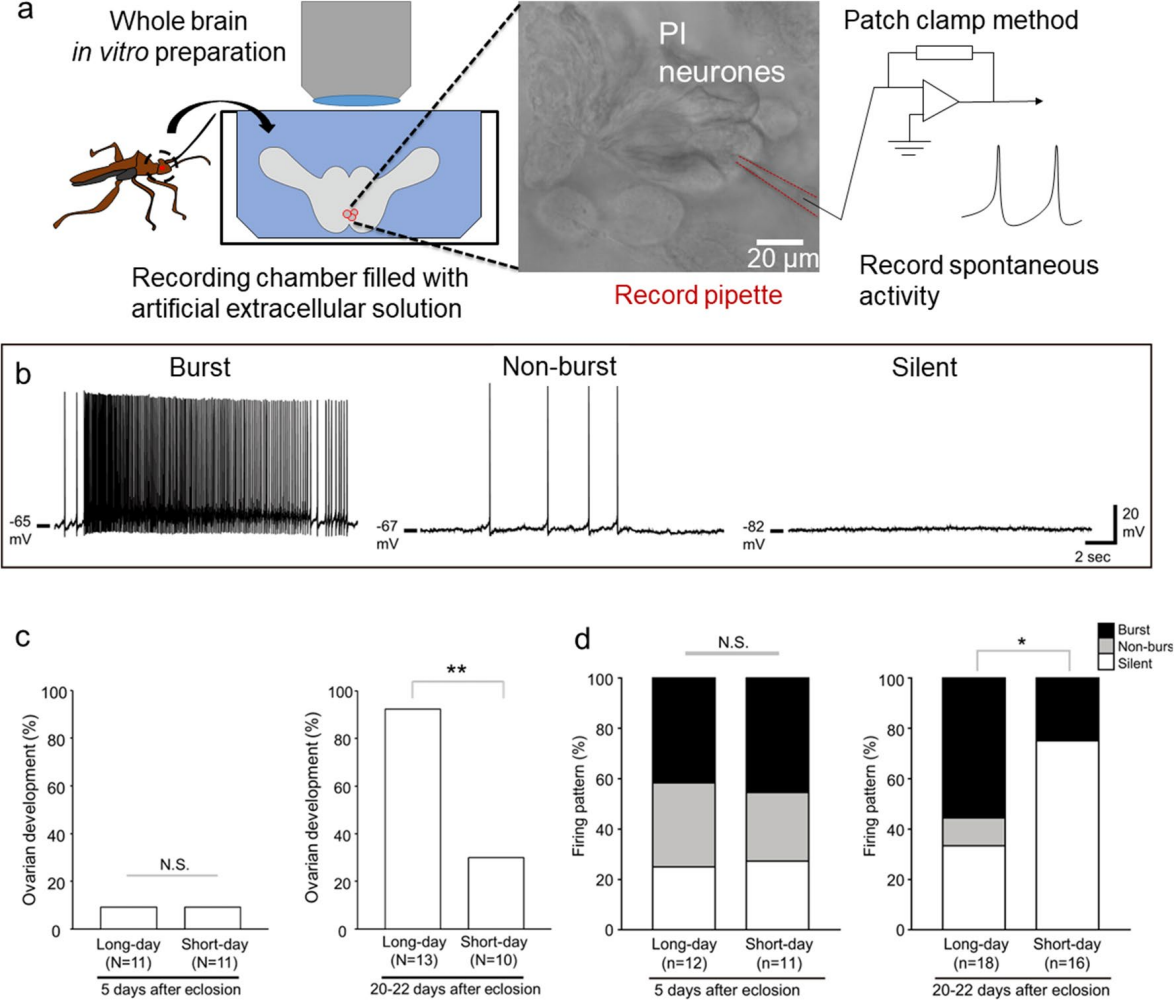
Photoperiodic neuronal response of pars intercerebralis (PI) neurones in *Riptortus pedestris*. **a** Conceptual illustrations showing neuronal activity recordings of PI neurones in whole-brain in vitro preparations in *R. pedestris*. **b** Representative traces showing burst (left), non-burst (middle), and silent (right) patterns in the PI neurones. **c**, **d** Columns showing proportions of ovarian development (**c**) and three firing patterns (**d**) in long- and short-day conditions 5 days after eclosion (left) and 20–22 days after eclosion (right). **c**, **d** χ^2^ test, *P < 0.05, **P < 0.01, N.S.: not significant. From Hasebe and Shiga (2021a) with permission

The bean bug *R. pedestris* show clear photoperiodic responses during reproduction; adult bugs enter reproductive diapause under short-day conditions and avert diapause under long-day conditions (Numata and Hidaka 1982). Surgical ablation of the PI reduces egg laying in females, suggesting that the PI plays an important role in promoting oviposition, although ovarian development is not affected (Shimokawa et al. 2008, 2014). Single-cell polymerase chain reaction (PCR) has revealed that PI neurones with large cell bodies express multiple neuropeptides, including insulin-like peptides (ILPs) and diuretic hormone 44 (DH44) (Hasebe and Shiga 2021a). The RNA interference-mediated knockdown of *Ilp*s and *Dh44* significantly reduced the number of eggs laid, suggesting that large PI neurones promote oviposition by releasing ILPs and DH44 (Hasebe and Shiga 2021a). In oviposition-promoting PI neurones, photoperiodic neuronal responses have been analysed using whole-brain in vitro preparations (Hasebe and Shiga 2021a) (Fig. 3a). PI neurones show various spontaneous activities, which were clarified into three types: ‘burst’, in which high-frequency burst activities were found; ‘non-burst’, in which spontaneous activities but not burst activities were found; and ‘silent’, in which multiple spontaneous firings were not found (Fig. 3b). In immature females, 5 days after eclosion, when ovaries were not developed on either long or short days, no significant differences were observed in the firing-pattern proportion in the PI neurones between the long and short days (Fig. 3c, d; Table 2). On the other hand, in mature females 20–22 days after eclosion, when ovarian development significantly varied between long and short days, PI neurones clearly changed their spontaneous firing activity according to the photoperiod; many PI neurones demonstrated high spontaneous firing (burst or non-burst) under long days, whereas many PI neurones were silent under short days (Fig. 3c, d; Table 2). This indicates that PI neurones upregulate their firing activity with ovarian maturation under long-day conditions, and promote oviposition by releasing oviposition-promoting neuropeptides.

A study by Hodková on PI in the linden bug *P. apterus* demonstrated multiple roles of the PI in the photoperiodic control of reproduction (Hodková 1976). In the linden bug, surgical ablation of the PI reduced oviposition regardless of the photoperiod. PI ablation also prevented short-day-induced inhibition of reproduction. These results suggest that both stimulatory and inhibitory centres exist in the PI of *P. apterus*. In *R. pedestris*, however, surgical ablation of the PI reduced oviposition but did not affect the short-day-induced inhibition of reproduction (Shimokawa et al. 2008, 2014). Based on previous reports and electrophysiological and genetic results by Hasebe and Shiga (2021a), the PI of *R. pedestris* may function as a photoperiodic stimulatory centre. In *R. pedestris*, an inhibitory centre may exist in other brain regions, such as the PL, because ablation of the PL averts reproductive arrest during short days (Shimokawa et al. 2008). Our future work will examine the photoperiodic shifts in the electrical activity of PL neurones.

### Pars intercerebralis neurones of *P. stali*

Photoperiodic effects on the electrical activity of PI neurones have also been analysed in another heteropteran species, *P. stali* (Hasebe and Shiga 2021b). *P. stali* shows photoperiodism in reproduction, similar to *R. pedestris*; the bug enters adult reproductive diapause in a short period (Kotaki 1987). Similar to *R. pedestris*, various firing activities (burst, non-burst, and silent) were observed in the PI neurones of *P. stali*. Interestingly, the photoperiodic neuronal responses of PI neurones in *P. stali* were contrary to those in *R. pedestris*. Many PI neurones in *P. stali* were silent under diapause-averting long days, and demonstrated high spontaneous activity under short days (Table 2). PI neurones in *P. stali* express multiple neuropeptides, including *Plauti stali*-myoinhibitory peptides (Plast-MIPs) (Hasebe and Shiga 2021b; Hasegawa et al. 2020). Plast-MIPs are neuropeptides that inhibit JH biosynthesis in the CC-CA complex and play an important role in inducing reproductive diapause under short-day conditions (Matsumoto et al. 2017; Tamai et al. 2019). Thus, in contrast to *R. pedestris*, PI neurones of *P. stali* may contribute to diapause induction by increasing neuronal activity and subsequent Plast-MIP release under short-day conditions.

### Transmission of photoperiodic information from clock cells to neurosecretory cells

Previous studies on *M. sexta*, *R. pedestris*, and *P. stali* suggested that NSCs in the PI and PL adequately alter their activity and subsequent neuropeptide release according to the photoperiod. This could be an important neuronal response in the neurosecretory system for photoperiodic control of physiological functions (Fig. 1). The next question is whether the neuronal response is controlled by the photoperiodic clock involving the circadian clock. Recently, a noteworthy study on *R. pedestris* demonstrated that the RNAi-mediated knockdown of the clock gene *period* (*per*) diminished the photoperiodic response of oviposition-promoting PI neurones (Hasebe and Shiga 2021a). In this bug, PER-immunoreactive cells are localised in close proximity to PDF cells at the anterior base of the medulla (Koide et al. 2021), and the removal of the anterior base region disrupts the photoperiodic response in reproduction (Ikeno et al. 2014). Based on these reports, it has been suggested that PI and PL NSCs may receive photoperiodic information from these upstream clock cells for photoperiodic control of PI and PL neuronal activities. Thus, the types of neural signals conveying photoperiodic information from clock cells to NSCs should be determined.

PDF might be the first prime candidate neural signal conveying photoperiodic information because it is a well-known neurotransmitter expressed in circadian pacemaker neurones in the proximal medulla of the optic lobe, classified as LNs, also called PDFMe neurones (Meelkop et al. 2011; Helfrich-Förster 1995). The PDF peptide is reportedly involved in circadian locomotor activity in various insects, including the fruit fly *D. melanogaster* (Renn et al. 1999; Helfrich-Förster et al. 2000), honeybee *Apis mellifera* (Beer et al. 2018), cockroach *Rhyparobia maderae* (Petri and Stengl 1997), and cricket *Gryllus bimaculatus* (Singaravel et al. 2003). Thus, PDF functions as an output molecule for circadian pacemaker neurones and is important for circadian rhythm formation in many insects.

In addition to its role in circadian rhythm formation, PDF has been extensively studied for its involvement in photoperiodic responses in several insect species. Besides the importance of PDF-immunoreactive neurones at the anterior base of the medulla in *P. terraenovae* and *R. pedestris* (Table 1), the PDF peptide itself was demonstrated, using genetic techniques (gene knockdown or knockout), to be important for response to photoperiodic change in *P. stali* (Hasebe et al. 2022) and for the critical day length of the photoperiodic response in *P. apterus* (Kotwica-Rolinska et al. 2022), as well as for the ordinary shallow photoperiodic response in starved *D. melanogaster* (Ojima et al. 2018).

Since these systemic gene knockdowns and knockouts disrupt PDF function in the whole body, it is necessary to further examine whether PDF signalling from circadian pacemaker neurones in the brain is important for photoperiodism. Neurosurgical and neuroanatomical studies on *P. terraenovae* have shown that PDF-immunoreactive s-LNv have synaptic connections with PL neurones and play an essential role in photoperiodic diapause control (Hamanaka et al. 2005; Shiga and Numata 2009). In *D. melanogaster*, neuroanatomical and cell-specific genetic modification analyses have revealed that PDF-positive s-LNvs connect with PI IPCs and are involved in the control of reproductive dormancy, and that PDF application gradually increases cAMP levels in the IPCs (Nagy et al. 2019). Based on these multifaceted studies, PDF may play an essential role in conveying photoperiodic information from circadian pacemaker cells to PI and PL neurones, which might underlie the photoperiodic control of reproduction, in various insects.

In association with the PDF, short neuropeptide F (sNPF) has been suggested to be involved in photoperiodic signalling. In *D. melanogaster*, a subset of *pdf*-expressing s-LNvs co-expresses *snpf* (Johard et al. 2009). The s-LNvs morphologically connect with the PI IPCs, and genetic repression of *snpf receptor 1* specifically in the PI IPC, reduces reproductive dormancy (Nagy et al. 2019). In addition, a single application of sNPF directly upregulates cAMP levels in PI IPCs, similar to the PDF peptide, and the co-application of PDF and sNPF results in stronger cAMP upregulation (Nagy et al. 2019). These findings suggest that sNPF is involved in photoperiod signalling in NSCs by cooperating with PDF signalling.

**Fig. 4:**
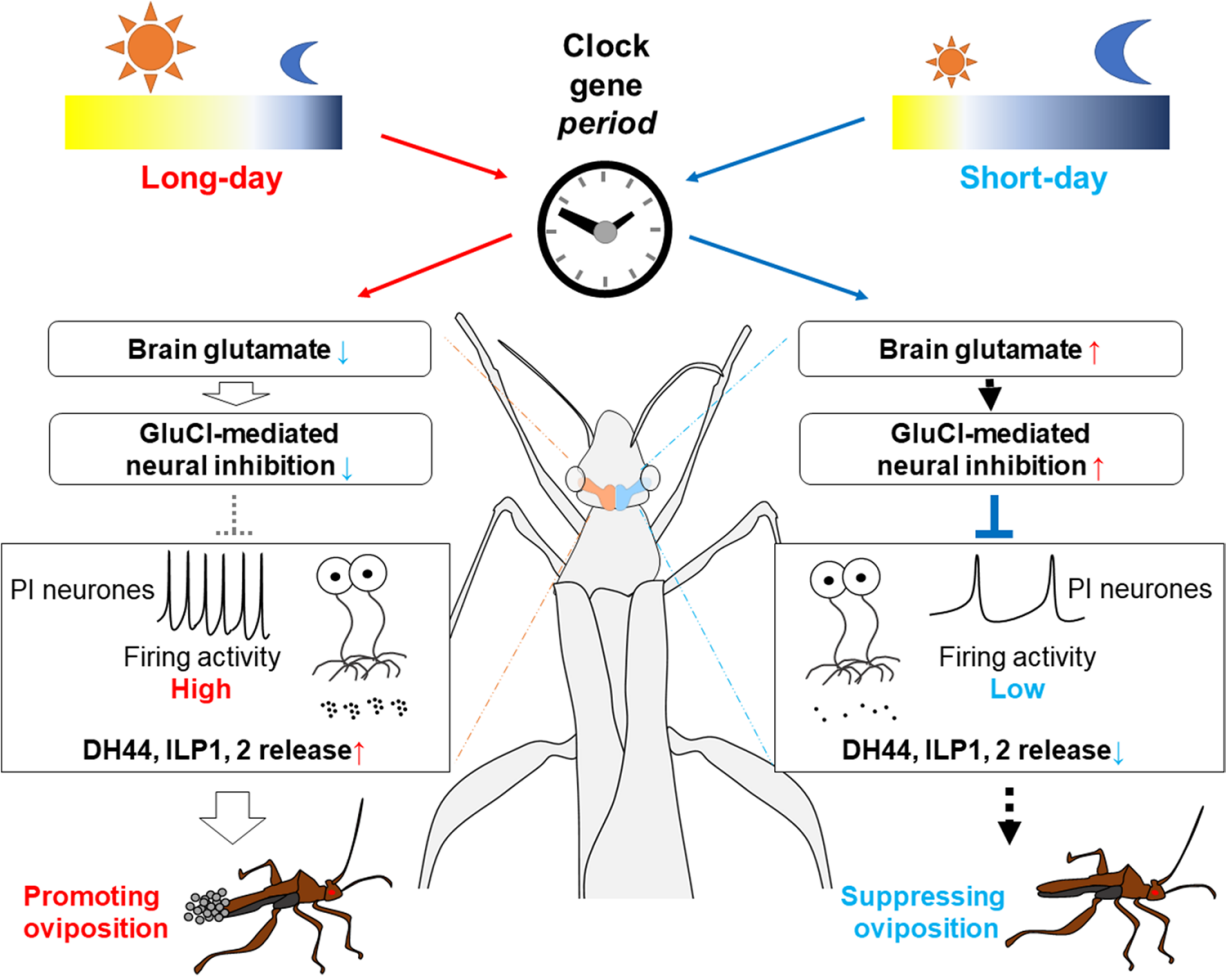
Predicted hierarchical pathway for photoperiodic oviposition control through glutamatergic signal—pars intercerebralis (PI) neurones in *Riptortus pedestris*. Extracellular brain glutamate levels photoperiodically change according to the clock gene *period* and upregulated under short days. Glutamate photoperiodically controls the neuronal activity of oviposition-promoting PI neurones via an inhibitory glutamate receptor, glutamate-gated chloride channel (GluCl), which contributes to photoperiodic control of oviposition. A conceptual illustration based on Hasebe and Shiga (2022) and Hidalgo and Chiu (2022)

Although analyses of neurotransmitters that convey photoperiodic information have mainly focussed on the PDF, it has also been reported that the PDF is not essential for photoperiodic signal transmission in a few species. In *R. pedestris*, RNA interference of *pdf* did not affect the photoperiodic response in reproduction, although ablation of the PDF cell region cancelled this effect (Ikeno et al. 2014). Mutation in *pdf* also did not diminish the photoperiodic change in ovarian dormancy in normally fed *D. melanogaster* (Nagy et al. 2019). Thus, other neurotransmitters convey photoperiodic information. Recently, Marteaux et al. performed a comprehensive gene knockdown of neurotransmitters known to be output molecules from clock cells in *R. pedestris* (Des Marteaux et al. 2022). Although photoperiodic diapause control was not affected by the knockdown of genes encoding neurotransmitters, such as DH31, sNPF, neuropeptide F, ion transport peptide, neuropeptide-like precursor 1, and the synthesis enzyme of acetylcholine, choline acetyltransferase, the knockdown of *vesicular glutamate transporter* (*vglut*), which brings glutamate into synaptic vesicles, tended to avert diapause under short-day conditions (Des Marteaux et al. 2022). Glutamate is a well-known classical neurotransmitter in the central nervous system (CNS) and is conserved from insects to vertebrates (Fonnum 1984; Pascual-Anaya and D'Aniello 2006; Bicker et al. 1988; Daniels et al. 2008), but it has not been studied as a neurotransmitter that conveys photoperiodic information. In *R. pedestris*, glutamatergic signals have been analysed in detail in the photoperiodic mechanism (Hasebe and Shiga 2022). Extracellular glutamate levels in the brain altered photoperiodically according to the *per* expression and were much higher under diapause-inducing short days (Fig. 4). Glutamate directly inhibits oviposition-promoting PI neurones through the glutamate-gated chloride channel (GluCl), an inhibitory glutamate receptor. Genetic suppression of glutamate-converting enzymes and *glucl* attenuates both the photoperiodic control of reproduction and the photoperiodic responses of PI neurones. These results clearly demonstrate that brain glutamatergic signals are photoperiodically regulated by the circadian clock and transmit photoperiodic information to oviposition-promoting PI neurones for photoperiodic control of oviposition (Fig. 4). As mentioned above, glutamate is a neurotransmitter that is widely conserved from insects to vertebrates. Thus, future research focussing on glutamate may shed light on determining conserved photoperiodic control mechanisms in a wide range of animal phyla.

The analysis of neural signals that transmit photoperiodic information from clock cells to NSCs is still limited, and neurotransmitters other than PDF, sNPF, and glutamate should be involved. As demonstrated in previous studies (Hasebe and Shiga 2021a, 2022), combining molecular genetics with physiological analyses at the brain and cellular levels would contribute to the elucidation of photoperiodic transmission in the integration mechanism shown in Fig. 1.

## Neural mechanisms underlying gastropod photoperiodism

### Seasonal regulation of neuroendocrine systems

After being perceived by photoperiodic photoreceptors, seasonal information is processed by photoperiodic clocks, similar to insects, evoking changes in the neuroendocrine system (Fig. 1), particularly in reproduction and growth. However, despite its importance, details of the photoperiodic regulation of neuroendocrine systems relevant to reproduction and growth have been studied in only a few molluscan species (McCrone and Sokolove 1986; Hamanaka and Shiga 2021). In reproduction and growth, the cerebral ganglion, which comprises the CNS (i.e. the brain), plays a crucial role in processing photoperiodic information to convey the resulting signals to key NSCs located in the ganglion, as highlighted in previous reviews (Wayne 2001; Flari and Edwards 2003). Nevertheless, few studies have addressed whether and how photoperiodic information alters the activity of NSCs for photoperiodic phenotypic changes such as gonadal maturation and egg laying.

In the giant garden slug *Limax maximus*, which shows a typical long-day response in gonadal maturation (Sokolove and McCrone 1978), the cerebral ganglia have been demonstrated to be responsible for mediating photoperiodic signals received by unknown photoperiodic photoreceptors to the reproductive glands (McCrone et al. 1981). Intriguingly, Sokolove et al. demonstrated that yet-to-be-identified cells (called M cells) in the parietal ganglia play a role in inducing egg laying in mature *L. maximus* in preliminary experiments (van Minnen et al. 1983). However, the series of studies ceased in the 1980s, and the nature of the gonadotropic hormone in the cerebral ganglia, as well as the cells producing the hormone, have not been elucidated. Thus, the physiological mechanisms underlying photoperiodism remain largely unknown in *L. maximus*.

A neurophysiological system of egg-laying behaviour in *A. californica* has been extensively investigated (Ferguson et al. 1989; Wayne 2001). Electrical signals initiated in the cerebral ganglia are transmitted to the nearby pleural ganglia and subsequently to bag cells in the abdominal ganglion (Ferguson et al. 1989). This pathway elicits a long-lasting bursting activity called afterdischarge of bag cells, which results in the release of egg-laying hormones (Wayne 2001). Unfortunately, sea hares mainly use seawater temperature as an environmental cue to define the breeding season, and the weak photoperiodic response makes it impractical to further investigate its effect on egg-laying behaviour (Wayne and Block 1992), which is briefly introduced in a later section.

### Photoperiodic control of egg laying in *Lymnaea stagnalis*

The freshwater pond snail *Lymnaea stagnalis* (Gastropoda, Basommatophora) displays photoperiodism in its egg-laying behaviour (Bohlken and Joosse 1982). However, the original report revealed the photoperiodic effect on egg laying only in group rearing conditions; thus, the precise proportion of ovipositing snails has been elusive. Importantly, a follow-up study explicitly demonstrated that egg laying in pond snails is photoperiodically controlled at the individual level. The cumulative proportion of egg-laying snails was higher in long-day conditions than in medium-day conditions of 12 h light and 12 h darkness with a critical day length between 12 and 13 h at 20 °C, although the gonad-somatic index did not change with photoperiod (Kitai et al. 2021).

### Ovulation and ovulation hormone

**Fig. 5:**
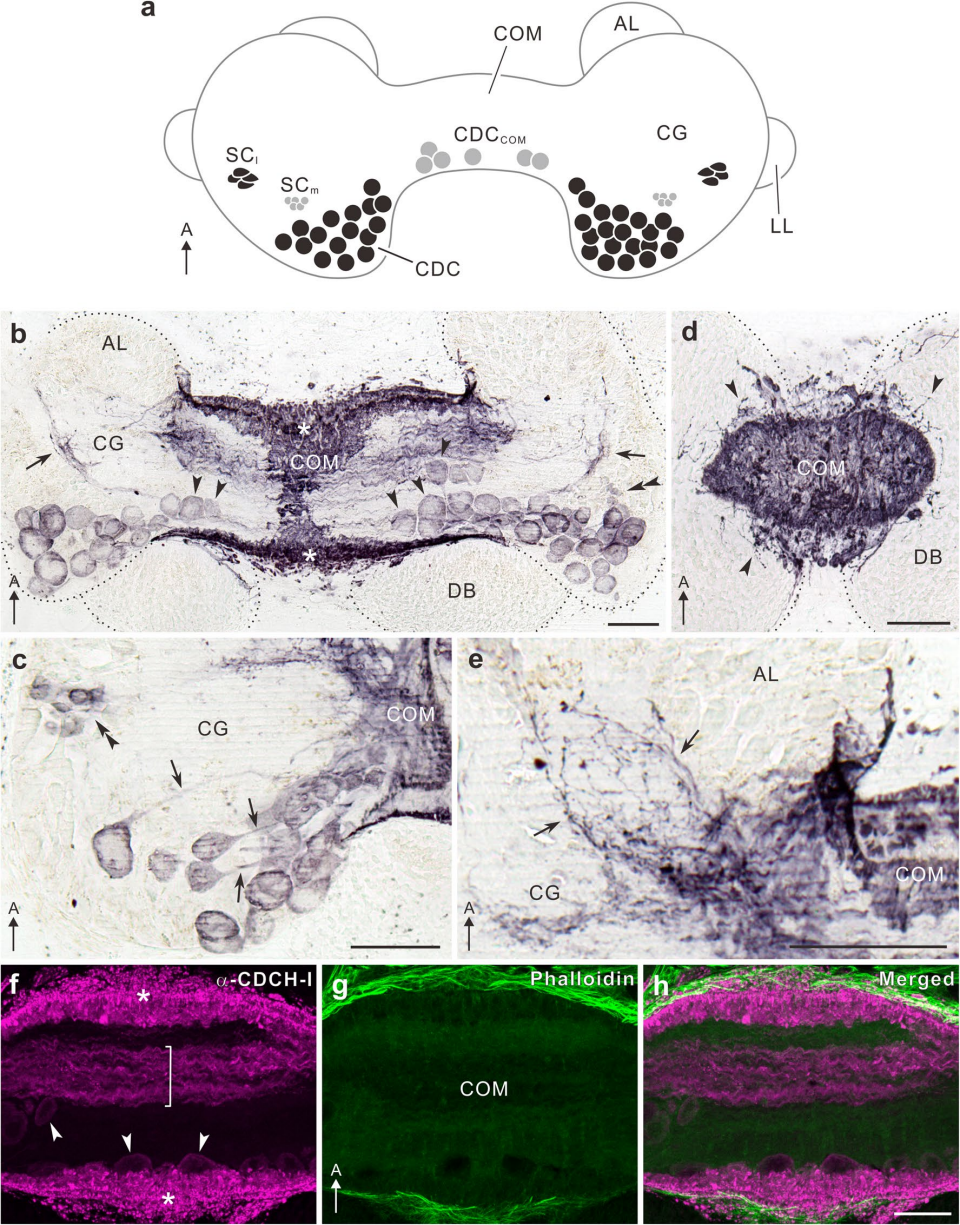
Distribution of caudo-dorsal cell hormone-I-immunoreactive neurones in the cerebral ganglia of the pond snail, *Lymnaea stagnalis*. **a** Schematic diagram depicting caudo-dorsal cell hormone-I-immunoreactive (CDCH-I-ir) cell bodies. Dorsal view (anterior points up). Grey cells represent two novel group of CDCH-I-ir cells described in Hamanaka and Shiga (2021), while black cells indicate classic CDCH-I-ir cells known before the account. **b**–**h** Horizontal sections of 40 μm thickness. **b**–**e** Immunoperoxidase labelling. **b** Large CDCH-I-ir cell bodies (CDCs) are located at a medio-posterior region of the cerebral ganglion (CG). Arrowheads indicate cell bodies inside the commissure (CDC_COM_). A cluster of small cell bodies (SC_m_, double arrowhead) was labelled anterior to the CDC cell bodies. **c** Horizontal section through a ventro-posterior region of the cerebral ganglion. Loosely clustered CDC cell bodies project their main axons towards the commissure (COM, arrows). A double arrowhead shows a cluster of small cells (SC_l_). **d** Horizontal section through a dorsal part of the commissure. Many short immunoreactive fibres invade part of the dorsal body (DB, arrowheads). **e** Horizontal section through part of the anterior lobe (AL). Some CDCH-I-ir neurites are sparsely distributed in a neuropil region lateral to the commissure (arrows). Black dotted lines indicate outer margin of the cerebral ganglia and dorsal body. **f–h** Immunofluorescence labelling of horizontal sections through the centre of the commissure. CDCH-I-immunoreactivity is shown in magenta (**f**) and Alexa 488-phalloidin labelling in green (**g**). Corresponding merged image in (**h**). In the periphery, many blebby immunoreactive fibres are distributed (asterisks in **f**). In the centre, a bundle of immunopositive fibres linking left and right cerebral ganglia is running (bracket). Arrowheads indicate immunopositive cell bodies within the commissure (CDC_COM_). *A* anterior, *LL* lateral lobe. Scale bars (**b**–**e**, **h**) 100 μm; **f**–**h** have the same scale. From Hamanaka and Shiga (2021) with permission

Egg laying in *L. stagnalis* is controlled by neurosecretory caudo-dorsal cells (CDCs), which are a bilaterally located group of neurones in the cerebral ganglia (Fig. 5) that resemble the bag cells in *A. californica* (Koene 2010). Ovulation, egg mass production, and stereotyped egg-laying-associated behaviours are initiated by synchronous long-lasting bursting activity, known as afterdischarge, lasting 30–60 min, and the subsequent release of an ovulation hormone or CDC hormone (CDCH) -I from these neurones (de Vlieger et al. 1980; ter Maat et al. 1986, 1989; Geraerts et al. 1991). CDCH-I is a peptide of 36 amino acids encoded by a single pre-prohormone that includes 11 distinct predicted neuropeptides in total (Hermann et al. 1997; Koene 2010). The importance of CDCs in egg laying was first discovered when snails with cauterised CDCs failed to lay egg masses (Geraerts and Bohlken 1976).

### Neuroanatomy of caudo-dorsal cells

**Fig. 6:**
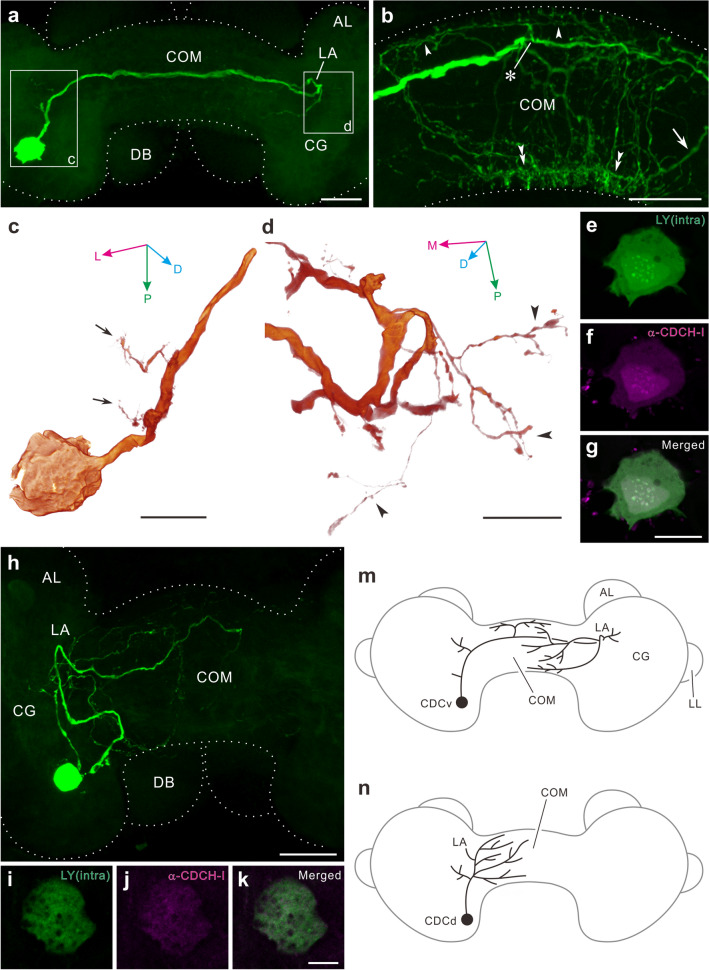
Morphology of a single caudo-dorsal cell in the pond snail, *Lymnaea stagnalis*. **a** Confocal stack of a single ventral caudo-dorsal cell (CDCv) filled with Lucifer Yellow. Ventral view (anterior to the top). CDCv sends a main axon towards the contralateral cerebral ganglion (CG) through the commissure (COM). The main axon returns to the commissure in an area called loop area (LA) on the contralateral side. **b** Enlarged image of the commissure. Arrowheads indicate fine varicose fibres derived from the thick main axon (asterisk), which continues to run towards the contralateral hemisphere. The axon which returns from the contralateral side (arrow) also bears many fine processes to form terminal arborisations at the periphery of the commissure (double arrowheads). **c**, **d** Three-dimensional reconstruction of the cell body and neurites in rectangles of panel (**a**). The main axon of CDCv forms laterally a few short extensions (arrows in **c**) in a neuropil area near the cell body. The contralateral axon also bears lateral extensions around the loop area (arrowheads in **d**). For orientation of view, refer to three-way arrows in panels. **e–g** CDCH-I immunoreactivity of the cell body of CDCv filled with Lucifer Yellow (single 4.8-μm-thick optical slices). Lucifer Yellow-filled cell body (green in **e**) exhibits CDCH-I immunoreactivity (magenta in **f**). Corresponding merged image in (**g**). **h** Confocal stack of a single dorsal caudo-dorsal cell (CDCd) filled with Lucifer Yellow. Ventral view (anterior to the top). The cell body of CDCd sends a primary axon, which bears many fine neurites. The neurites of CDCd are intermingled in the neuropil of the ipsilateral cerebral ganglion to project towards the commissure. **i–k** CDCH-I immunoreactivity of the cell body filled with Lucifer Yellow (single 4.8-μm thick optical slices). The Lucifer Yellow-filled cell body (green in **i**) exhibited CDCH-I immunoreactivity (magenta in **j**). Corresponding merged image in (**k**). **m**, **n** Schematic diagram of two types of CDCs. Dorsal view (anterior to the top). **m** Cell body of CDCv extends its primary axon towards the contralateral cerebral ganglion, which bears some processes terminating on the periphery of the commissure. The axon in the contralateral side returns towards the commissure to form terminal arborisations. **n** Cell body of CDCd extends the main axon, which bears many processes on the ipsilateral side projecting to the commissure. A pair of dorsal bodies surmounting the cerebral ganglia and commissure is omitted for clarity. Dashed lines in panels (**a**, **b**, **h**) indicate outlines of the cerebral ganglion, dorsal body, and commissure. AL, anterior lobe; L, lateral; LL, lateral lobe; D, dorsal; M, medial; P, posterior. Scale bars (**a**, **b**, **h**) 100 μm, (**c**, **d**, **g**) 50 μm, (**k**) 20 μm; **i**–**k** and **e**–**g** have the same scale, respectively. From Hamanaka and Shiga (2021) with permission

The anatomical terminology for CDCs originates from their topological characteristics in the cerebral ganglia (Joosse 1964). Gomori staining revealed approximately 100 cells in the two hemispheres of the cerebral ganglia (Joosse 1964). However, an anti-CDCH-I antiserum identified 80–130 cells per hemisphere (Fig. 5a–c); the right side always dominated (Hamanaka and Shiga 2021). In addition, CDCH-I-positive small cells medial (SCms) were found in the cerebral ganglia and CDCH-I cells in the commissure (an intercerebral neurohemal site) (Fig. 5b, c). The canonical CDCs are of two morphological subtypes, viz. ventral and dorsal CDC (CDCv and CDCd); both types of CDCs form varicose terminal arborisations on the periphery of the commissure (Fig. 6a, b) (Joosse 1964; de Vlieger et al. 1980; van Minnen et al. 1988; Hamanaka and Shiga 2021). CDCv projects the main axon into the commissure that interconnects clusters of CDCs in both hemispheres (de Vlieger et al. 1980) and bears characteristic lateral branches (Fig. 6a, c, d, m), whereas CDCd neurites are restricted only to the ipsilateral side (Fig. 6h, n).

In addition to ablation experiments on CDCs (Geraerts and Bohlken 1976), a later study demonstrated that the injection of highly purified or synthetic CDCH-I is capable of inducing egg laying (ter Maat et al. 1989). These findings strongly imply that either failure or reduction in CDCH-I release from CDCs could cause photoperiodic suppression of egg laying under short- or medium-day conditions.

### Photoperiodic regulation of caudo-dorsal cells

**Fig. 7:**
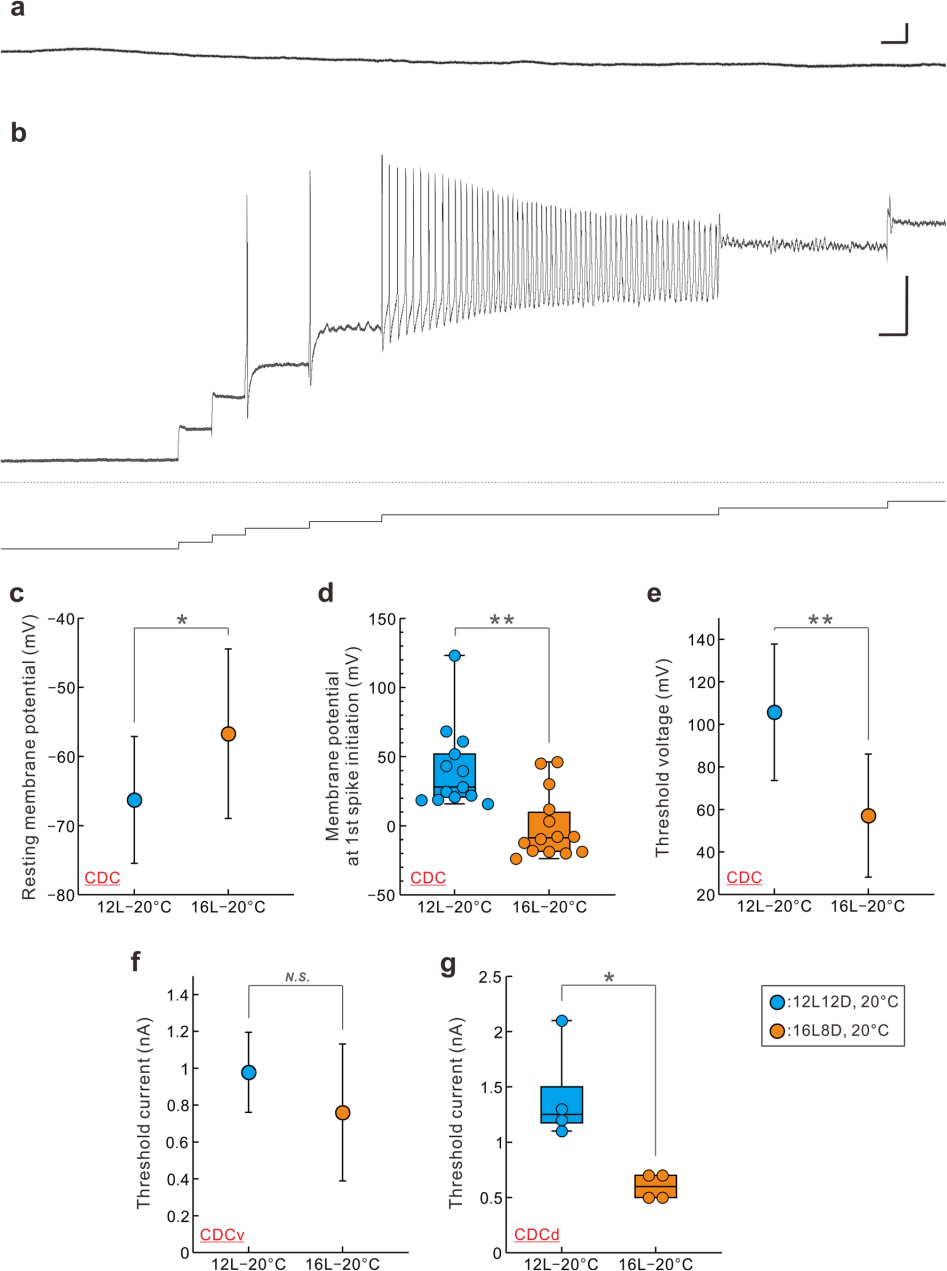
Intracellular recording and electrophysiological properties of caudo-dorsal cells in the pond snail, *Lymnaea stagnalis*. Intracellular recording from a ventral caudo-dorsal cell (CDCv). Intracellular recording was performed from the cell body of a CDCv of a 25-week-old snail reared at 20 °C under long-day conditions (16L8D). **a**, **b** The resting membrane potential for this particular neurone was – 42.5 mV (**a**). It does not show spontaneous action potentials, but the first spike was evoked by injection of positive direct current of + 0.3 nA (**b**). Current was applied by a 0.1 nA step (see the bottom trace of Panel **b**). In this neurone, continuous injection of + 0.5 nA current triggered bursting neuronal activity (discharge) typical of CDCs. **c–g** Comparison of electrophysiological properties of CDCs between medium-day and long-day conditions, which include resting membrane potential (**c**), membrane potential at 1st spike initiation (**d**), threshold voltage (**e**), threshold current (**f**, **g**). The comparison of threshold current was made in a cell-specific manner (CDCv in (**f**), CDCd in (**g**)). Graphs represent either average (circle) ± standard deviation (**c**, **e**, **f**) or box-plot indicating median, interquartile ranges, and maximum and minimum values (**d**, **g**). Circles in box-plots indicate the value of each sample. *P < 0.05; **P < 0.01 [unpaired two-tailed *t* test (**c, f**), Mann–Whitney *U* test (**d**, **e**, **g**)]. Scale bars (**a**) 5 s (horizontal) 10 mV (vertical), (**b**) 1 s (horizontal) 20 mV (vertical). From Hamanaka and Shiga (2021) with permission

Intracellular recordings have demonstrated that the excitability of CDCs changes in a photoperiod-dependent manner; it is higher in long-day than medium-day conditions (Hamanaka and Shiga 2021, Fig. 7). CDCs do not display spontaneous spiking activities in the resting state; they are preceded by an inhibited state and followed by an active state; however, the injection of a train of current pulse stimuli into CDCs in the resting state can induce long-lasting afterdischarge (Kits 1980). The steady injection of a positive current also induced action potentials and/or bursting activities (Fig. 7a, b), and seven electrophysiological properties of CDCs were compared between long- and medium-day conditions (Hamanaka and Shiga 2021). The resting membrane potential was shallower under long- than under medium-day conditions (Fig. 7c). The membrane potential at which the first spike was initiated was higher under medium-day conditions (Fig. 7d), and accordingly, the threshold voltage was higher under medium-day conditions than under long-day conditions (Fig. 7e). The photoperiodic difference in the threshold current (Fig. 7f-g), which is required to elicit action potentials, was cell type-specific. No significant difference was observed in the threshold current of CDCv (Fig. 7f), whereas CDCd was less active under medium- than under long-day conditions (Fig. 7g) (Hamanaka and Shiga 2021). Electrophysiological properties indicate that CDC excitability is higher under long-day conditions than under medium-day conditions, although the underlying physiological mechanisms remain to be elucidated.

The expression levels of *cdch* mRNA in the cerebral ganglia were also higher under long- than under medium-day conditions (Kitai et al. 2021). These results suggest that *cdch*-expressing CDCs receive photoperiodic inputs that increase *cdch* expression and the excitability to release more CDCHs in response to long days.

### Canopy cells under photoperiodic regulation in *L. stagnalis*

**Fig. 8:**
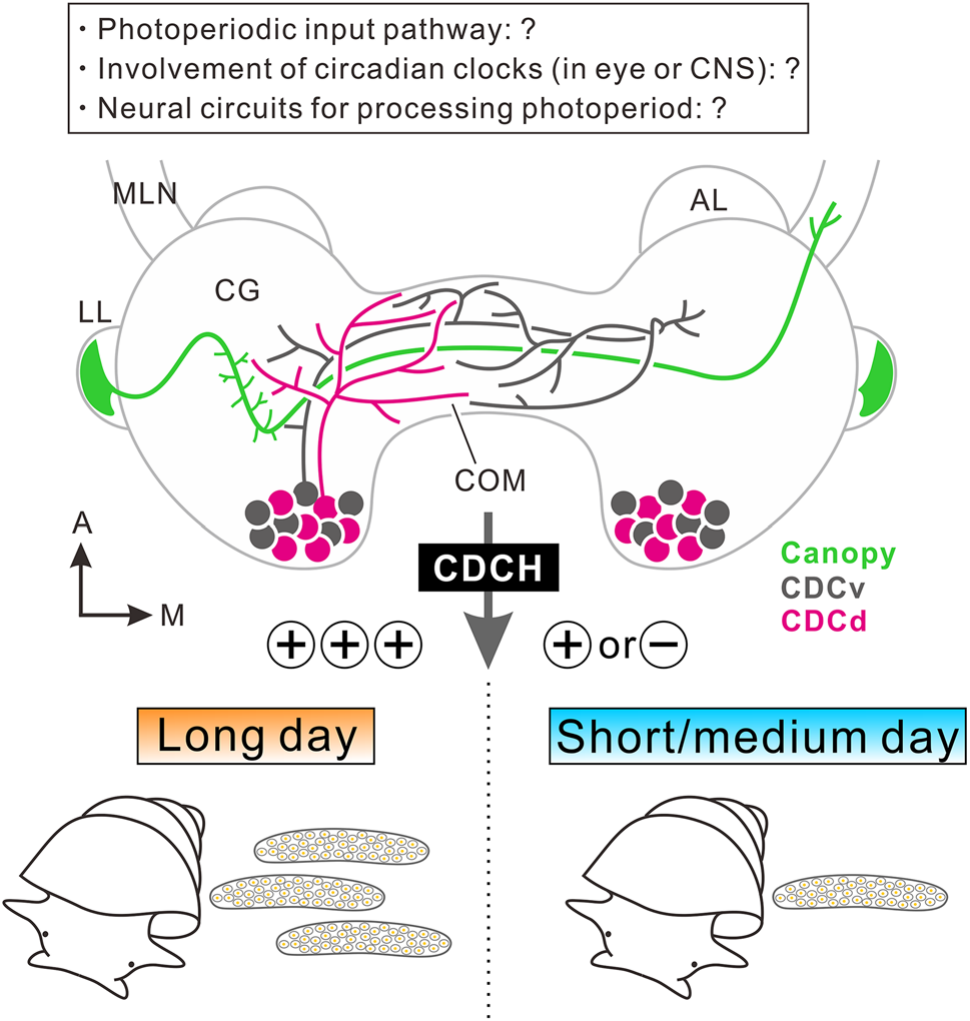
Schematic illustration of neurosecretory cells under photoperiodic regulation in *L. stagnalis*. Projection patterns of caudo-dorsal cells (CDCs) and a canopy cell, whose cell bodies are located in the left cerebral ganglion (CG), are described for clarity. Long days stimulate release of CDC hormone (CDCH) to promote egg laying or short days suppress its release in order to inhibit egg laying. *A* anterior, *AL* anterior lobe, *CDC* caudo-dorsal cell, *CDCd* dorsal CDC, *CDCv* ventral CDC, *COM* commissure, *LL* lateral lobe, *M* medial, *MLN* median lip nerve. From Hamanaka and Shiga (2021) with permission

Another type of NSC under photoperiodic control is the neurosecretory canopy cell. Canopy cells are located in the lateral lobe, the budding structure of the cerebral ganglion (Fig. 5a; Lever and Joosse 1961; Brink and Boer 1967). The functional role of neurones remains unknown; however, quantitative electron microscopy has revealed that the number and volume of subcellular organelles responsible for the production of secretory materials are larger under long- than under short-day conditions (van Minnen and Reichelt 1980). A recent electrophysiological study also demonstrated that the activity of canopy cells is higher under long- than under medium-day conditions (Hamanaka and Shiga 2023). The canopy cell forms putative terminal arborisations on the median lip nerve (Fig. 8) and produces a molluscan insulin-like peptide (van Minnen et al. 1979, 1989; Benjamin et al. 1980). According to its neurosecretory characteristics, the canopy cell appears to release its secretory materials into the haemolymph in a photoperiod-dependent manner.

What mechanisms control the activity of CDCs and canopy cells during different photoperiods? Fig. 8 illustrates the two types of NSCs under photoperiodic control. It is necessary to identify and characterise the upstream local circuits that process and integrate photoperiodic information. Combined with a previous study on *L. maximus* (McCrone et al. 1981), the cerebral ganglia may play an important role in the processing of photoperiodic information in snails. In the photoperiodic mechanism, the circadian clock is considered to play a crucial role in time measurement before submitting day-length information to NSCs (Fig. 1, Nelson et al. 2010). Understanding the circadian clock system in snails is indispensable; whether its involvement in photoperiodism is also present in molluscan species remains unclear. We summarised circadian rhythm and circadian clock systems in the following paragraphs for future reference to help to explore a causal link between photoperiodism and circadian clock systems.

### Circadian rhythm and circadian clock system

Circadian rhythms of locomotion have been demonstrated in a number of gastropods, including *A. californica* (Kupfermann 1968; Jacklet 1972), the cloudy bubble snail *Bulla gouldiana* (Block and Davenport 1982), the freshwater snail *Helisoma trivolvis* (Kavaliers 1981), the garden snail *Helix aspersa* (Bailey 1981), *L. maximus* (Sokolove et al. 1977), the littoral gastropod *Melanerita atramentosa* (Zann 1973), the nudibranch *Melibe leonina* (Newcomb et al. 2004, 2014), the tropical freshwater snail *Bulinus tropicus* (Chaudhry and Morgan 1983), and the tropical snail *Melanoides tuberculata* (Beeston and Morgan 1979). Circadian rhythm of oxygen consumption has been reported in the salt marsh periwinkle *Littorina irrorata* (Shirley and Findley 1978). It is noteworthy that in the frilled sea hare *Bursatella leachi plei*, only a daily locomotion rhythm has been demonstrated so far, with ambiguous evidence of the circadian rhythm (Block and Roberts 1981). In *L. stagnalis*, diurnal and circadian rhythms in locomotion have been demonstrated (Wagatsuma et al. 2004), with faint evidence of diurnal rhythmicity in back-swimming speed (Aono et al. 2008).

In *A. californica*, *B. gouldiana*, and *B. leachi plei*, the isolated eyes and retinal neurones display a circadian rhythm in terms of the frequency of spontaneously occurring optic nerve impulses (Jacklet 1969; Block and Roberts 1981; Block and Wallace 1982; Michel et al. 1993). These studies imply that the eyes are the site of at least one circadian pacemaker, which is supported by the anatomical localisation of cells expressing *per* and PER in the eyes (vide infra). The eyes are known to play an essential role in the integrity of the circadian locomotor rhythm in *B. gouldiana*, as eyes-ablated animals display disorganised locomotor activity (Block and Davenport 1982). In contrast, in *A. californica*, eyes-ablated animals, some of which fail to show a free-running rhythm and others display a free-running rhythm in constant darkness, are still capable of responding to a light–dark cycle. Thus, the eye is neither the only photoreceptor nor the only circadian oscillator coupled to the locomotor rhythm (Block and Lickey 1973; Lickey et al. 1977). In *A. californica*, the eye appears to be one of several endogenous oscillators coupled with locomotion and extraocular photoreceptors are capable of controlling this behaviour. In *B. leachi plei*, which displays an ambiguous circadian rhythm in locomotion, the eyes play a less prominent role than the eyes of *Aplysia* in controlling the activity rhythm (Block and Roberts 1981). Thus, the importance of the eyes for generating the circadian locomotor rhythm seems to be species-specific, and the characterisation of clock cells outside the eye and extraocular photoreceptors is required.

### Location of circadian pacemaker cells in the eye

The anatomical localisation of putative circadian pacemakers in the eye has been accomplished in only two gastropods, namely *A. californica* and *B. gouldiana*, using immunohistochemistry with an antibody raised against a conserved region of *D. melanogaster* PER protein (Siwicki et al. 1989). In *A. californica*, cell bodies in the outer layer of the retina and fibres in the optic nerve exhibited PER immunoreactivity, and immunopositive signals were detected in the cytoplasm of the cell bodies. Western blotting has shown that the levels of this antigen in the eyes exhibit distinctive diurnal fluctuations in *A. californica* (Siwicki et al. 1989). In *B. gouldiana*, intense PER immunoreactivity was observed in the cell bodies of basal retinal neurones, with signals restricted to the cytoplasm, as in *A. californica*, whereas no fibres in the optic nerve were immunolabelled with the anti-PER antibody (Siwicki et al. 1989).

Furthermore, in situ hybridisation confirmed *per* expression in basal retinal neurones and photoreceptors of the retina in *B. gouldiana* (Constance et al. 2002). Interestingly, by quantitative in situ hybridisation, the authors found a distinctive diurnal oscillation in *per* expression in basal retinal neurones, whereas they failed to demonstrate a circadian change in *per* expression under constant conditions between two circadian time points (CT5 and CT13) that corresponded to the peak and trough time points under a light–dark regime, respectively (Constance et al. 2002). Since western blotting of the eyes did not detect a diurnal oscillation in PER levels (Constance et al. 2002), the oscillation occurred only at the mRNA level restricted to the basal retinal neurones, but not at the protein level under light–dark conditions in *B. gouldiana*.

### Location of putative circadian pacemaker cells in the CNS

In the CNS of *M. leonina*, fluorescent in situ hybridisation labelled putative circadian clock cells that simultaneously expressed four circadian clock genes, *clock* (*clk*), *per*, *non-photoreceptive cryptochrome* (*npcry*), and *photoreceptive cry* (*pcry*), which consisted of two bilateral cells in the cerebropleural ganglia and a group of < 10 neurones in each pedal ganglion (Duback et al. 2018). Furthermore, the oscillation of these clock genes was investigated throughout the course of a day by quantitative PCR; both *per* and *pcry* displayed a significant variance in expression, whereas neither *clk* nor *npcry* did. For all clock genes, a tendency for peak expression around zeitgeber time 18 and low expression during the daytime was observed (Duback et al. 2018). These data suggest the presence of circadian pacemaker cells in the CNS of *M. leonina*, which may be involved in the generation of circadian locomotor activity.

### Relationship between the circadian clock and photoperiodism

In *A. californica*, a cluster of bag cells in the abdominal ganglion is responsible for egg laying in this species (Ferguson et al. 1989). Unfortunately, the photoperiodic effect on egg laying in sea hares is not as robust as that of temperature. However, under warm temperature conditions, short days (8 L:16D) have been shown to stimulate the frequency of egg laying compared to long days (16L:8D) (Wayne and Block 1992). This weak stimulatory effect of short days occurred only in animals captured from summer to autumn, corresponding to their natural breeding seasons in the field. Eye removal experiments suggest that the eyes play a role in photoperiodism in *A. californica* because bilaterally eye-ablated individuals exhibit no photoperiodic response at all (Wayne and Block 1992).

In *A. californica*, the eyes harbour a circadian pacemaker that is responsible for generating the free-running rhythm of the frequency of optic nerve impulses and partially for locomotion (Jacklet 1969; Block and Lickey 1973; Lickey et al. 1977). Thus, it remains to be resolved whether the observed loss of photoperiodism in eye-ablated animals is attributable to a lack of photoperiodic perception, loss of circadian pacemakers, or both. Photoperiodic information received by the eyes (Fig. 1) may be conveyed to bag cells through the optic nerve to modulate the frequency of egg laying. Since this research, the investigation of the photoperiodic regulation of egg laying in *A. californica* has been suspended, and thus, the following study on the photoperiodic control of bag cells should be conducted. The location of the circadian clock in *L. stagnalis* is an intriguing question. The identification and characterisation of clock neurones, possibly either in the eyes or CNS (Fig. 8), may shed light on the comprehension of photoperiodic systems in snails. Therefore, it is important to examine the relationship between clock neurones and CDCs.

## Commonalities and uniqueness of insects and snails, and future perspectives

Both insects and snails require a certain time period for photoperiodic perception and recognition, suggesting a similar brain mechanism underlying this response. Functionally similar NSCs have been identified in photoperiodic mechanisms that control seasonal egg laying. ILP/DH44 producing PI neurones in the insect *R. pedestris* and CDCH producing CDCs in the snail *L. stagnalis* promote egg laying under long days, and their electrical excitability is attenuated to reduce oviposition under short or medium days. NSCs of both ILP/DH44-PI neurones and CDCs play a role in promoting egg laying. Other NSCs must play inhibitory roles in reproduction. The induction of photoperiodic diapause or reproductive suppression is an active process requiring a number of short or medium days, in contrast to gonadal maturation or egg laying, which seems to proceed as a default function without environmental cues. Recently, in *D. melanogaster*, DH31-expressing neurones in the PL were found to be dormancy-inducing NSCs (Kurogi et al. 2023). PL neurones are another type of NSCs that negatively control insect reproduction (Table 1). They are likely to be activated under short days. Comparing the electrical activities of DH31-expressing PL neurones between short and long days in photoperiodic insects such as *P. terraenovae* and *R. pedestris* would be interesting. It would also be interesting to determine the effects of glutamate on PL neurones. Glutamate upregulation during short days (Fig. 4) may result in the direct or indirect promotion of the PL neuronal activity. In snails, some inhibitory neurones in egg laying or reproduction could be important under medium- to short-day photoperiods; future studies are warranted for clarification of the inhibitory cells or brain regions.

In the photoperiodic clock and counter mechanism, only the involvement of circadian clock genes or cells and potential clock cell networks has been demonstrated in insects; however, how clock cell networks achieve day-length measurement and day-number integration remains unknown. Recent knowledge of clock gene expression in the CNS and circadian behaviour in snails should promote an understanding of the circadian clock system. Analysis of the circadian clock involvement in the snail’s photoperiodic mechanism should be conducted. We presume that neuropeptides in the NSCs vary between insects and snails, as their behaviours are very different. However, clock genes, clock cell transmitters, and their functions in the photoperiodic mechanism may share commonalities between the two animal groups. Owing to its universal seasonal adaptation mechanism, the photoperiodic mechanism has evolved as part of the circadian clock system from the common ancestor. Knowledge of the link by glutamate between the circadian clock system and NSCs, and of circadian clock cell transmitters in insects may help explore the upstream mechanisms of neurosecretory CDCs in the photoperiodic mechanism of the snail *L. stagnalis*.

The molluscan CNS is much simpler than the insect brain. For instance, the number of neurones constituting the CNS of the hermaphroditic pond snail *L. stagnalis* is approximately 15,000 (Geraerts et al. 1991) or 20,000 (Kemenes and Benjamin 2009), which is as little as one-tenth of the brain of the model species, the fruit fly *D. melanogaster* (with ~ 200,000 neurones) (Raji and Potter 2021). In addition, the neurones themselves are relatively large, which makes it easier to monitor their activity at a single-cell level. Although relatively little information exists, the numerical advantage makes molluscan species ideal model organisms for the study of the underlying neural mechanism of photoperiodism.

Understanding how the photoperiodic clock discriminates between long and short days and how the photoperiodic counter counts the number of circadian clock cycles would be feasible in invertebrates with a simple CNS. These two questions are neurobiologically important in the photoperiodic mechanism. These interesting questions should be addressed in the near future.

## Data Availability

Relevant data are provided on your request.
